# Characterization of a novel sarcoma cell line with an EWSR1::POU2AF3 fusion

**DOI:** 10.3389/pore.2025.1611986

**Published:** 2025-03-11

**Authors:** Hannah Schwab, Maximilian Kerkhoff, Pauline Plaumann, Stéphane Collaud, Uta Dirksen, Dirk Theegarten, Thomas Herold, Stavros Kalbourtzis, Servet Bölükbas, Balazs Hegedüs, Luca Hegedüs

**Affiliations:** ^1^ Department of Thoracic Surgery, University Medicine Essen – Ruhrlandklinik, Essen, Germany; ^2^ Pediatrics III, West German Cancer Center, University Medicine Essen, Essen, Germany; ^3^ German Cancer Consortium (DKTK), Partner Site Essen, Essen, Germany; ^4^ National Center for Cancer diseases (NCT-West), Essen, Germany; ^5^ Department of Thoracic Surgery, Cologne Merheim Hospital, University of Witten/Herdecke, Cologne, Germany; ^6^ Institute of Pathology, University Medicine Essen, Essen, Germany

**Keywords:** sarcoma, chemotherapy, EWSR1, POU2AF3, patient derived cell line

## Abstract

Sarcomas with an EWSR1::POU2AF3(COLCA2) fusion are a very recently described entity of preferentially sinonasal origin and with undifferentiated round/spindle cell morphology. We established a novel cell line (PF1095) carrying a EWSR1::POU2AF3 fusion from the malignant pleural effusion of a 25-year-old sarcoma patient. The patient was first diagnosed with poorly differentiated neuroendocrine carcinoma based on tumor cell morphology and positivity to markers such as EMA, synaptophysin, and CD56. Later, the EWSR1 translocation was identified in the tumor cells with unknown partners and the patient received chemotherapy according to the Ewing 2008 protocol in combination with surgery and proton beam radiotherapy. At the time of cell line establishment, the disease progressed to pleural sarcomatosis with pleural effusion. In the cell line, we identified POU2AF3 as a fusion partner of EWSR1 and a TP53 frameshift deletion. Next, we determined the sensitivity of PF1095 cells to the currently approved chemotherapies in comparison to two conventional Ewing sarcoma lines (EW-7 and MHH-ES1) with the two most frequent EWSR::FLI1 fusions. Finally, we tested potential new combination therapies. We performed cell viability, proliferation, and cell cycle assays. We found that the proliferation rate of PF1095 cells was much slower than the EWSR1::FLI1 fusion lines and they also had a lower sensitivity to both irinotecan and doxorubicin treatment. Expression level of SLFN11, a predictor of sensitivity to DNA damaging agents, was also lower in PF1095 cells. Combination treatment with the PARP inhibitors olaparib and irinotecan or doxorubicin synergistically reduced cell viability and induced cell death and cell cycle arrest. This unique cell model provides an opportunity to test therapeutic approaches preclinically for this novel and aggressive sarcoma entity.

## Introduction

Small round cell sarcomas form a rare, heterogeneous, and highly aggressive group of tumors of soft tissue and bone that are categorized based on histomorphological similarities. They are associated with very poor outcome, especially in metastatic disease [1, 2]. Their etiology is still under investigation as tumor cells carry markers associated with both mesenchymal stem cells and neuroectodermal lineage [3–6]. The prototype of the characteristic histomorphology of small round cell sarcomas is the Ewing Sarcoma [1, 4], which is exemplary defined by the chromosomal translocation of an FET gene family member with an ETS transcription factor, mostly EWSR1::FLI1 [7, 8]. This gene fusion not only confirms the diagnosis but is also critical for the tumorigenesis [7, 9] as it is a driver mutation that encodes for a transcription factor with hundreds of potential target genes involved in the regulation of cell proliferation, cell differentiation, cell-cycle control, angiogenesis, and apoptosis [3, 5]. In Ewing sarcoma (ES), cell surface markers such as CD99, Fli-1, and caveolin-1 are used in diagnostics, while glycosphingolipid ganglioside antigen G (D2) (GD2) can serve as a possible target for anti-GD2 monoclonal antibody therapy [3, 10, 11]. The somatic mutation rate in Ewing sarcomas is low. The most frequent mutations affect the STAG2, CDKN2A, TP53, EZH2, BCOR, and ZMYM3 genes [12]. This emphasizes the importance of the characteristic FET::ETS translocation for tumorigenesis.

Historically, round cell sarcomas are classified as Ewing-like sarcomas if they harbor a fusion between EWSR1 and another transcription factor [5]. In the 5th edition, the WHO classification of soft tissue and bone tumors CIC-fused sarcomas, BCOR-rearranged sarcomas, and round cell sarcomas with EWSR1::non-ETS fusion are distinguished. Each of them is recognized as a specific entity, presenting specific genetic, morphologic, and clinical features [3, 13–15]. To classify the sarcomas correctly, a combination of morphologic, immunohistochemical, and molecular findings must be considered [4, 15].

The translocation found in the investigated PF1095 cell line consists of the FET gene family member EWSR1 and POU2AF3 (also called COLCA2). POU2AF3 was first described as a potential colorectal cancer predisposition gene [16, 17]. Later, increased expression of COLCA1/COLCA2 was found to be associated with increased primary biliary cholangitis susceptibility [18]. Recently, it was shown to play a role in the development of tuft cell lineage [19]. Tuft cells are chemosensory cells that are present in mucosal epithelial tissues, including the gastrointestinal track, and in the respiratory epithelium. They give rise to a variant of small-cell lung cancer (SCLC-P) as well. A major regulator of both normal tuft cell development and malignant transformation is the POU transcription factor POU2F3. POU2AF2 and POU2AF3 (COLCA2) are also required for these processes, and they serve as coactivators. They can bind DNA octamer motifs and class II POU transcription factors. POU2AF3 expression was detected in the human bronchial epithelium and SCLC-P tumors as well.

So far, there are just a few published cases of sarcomas where an EWSR and POU transcription factor family member fusion was identified as an oncogenic transcription factor. Yamaguchi et al. described a bone tumor with a fusion between EWSR1 and POU5F1 [20] and Sankar et al. presented an Ewing sarcoma-like tumor harboring a translocation creating an EWSR1::POU5F1 protein that functioned as an aberrant oncogenic transcription factor. They also questioned the classification of this tumor as a Ewing sarcoma by expressing the need for further investigations [5]. Recently, it has been proposed that sarcomas harboring a fusion of POU2AF3 with EWSR1 form a novel entity that often originate from the head and neck region, especially from the sinonasal track. They show an aggressive, highly metastatic behavior. They have a mixed round and spindle cell morphology and an unusual immunophenotype, often presenting a mixture of neuroendocrine or epithelial markers [16, 21, 22].

The treatment of Ewing and Ewing-like sarcomas follows a multimodal strategy through the combination of surgical resection and/or local radiotherapy and multiagent (induction) chemotherapy [3, 11]. The long-term survival of patients with localized Ewing sarcomas is 70%, however, patients who develop metastasis or experience tumor relapse have a poorer prognosis [23]. Additionally, the current treatment regimen is associated with significant long-and short-term side effects for survivors [24].

Ewing-like sarcomas contain different gene fusions and rearrangements to Ewing sarcomas, however, so far, no specific therapeutic target has been identified in these tumors. For this reason, most patients are treated according to the Ewing sarcoma protocol and enrolled in Ewing sarcoma trials, since clinical trials for Ewing-like sarcomas are rarely available [11, 25]. It was even proposed that small round cell sarcomas with distinct fusions and rearrangements should be considered as separate entities and that separate therapeutic trials separately should be performed [9]. There is also a difference in the outcome between the different undifferentiated small round cell sarcomas subtypes, for instance, patients with CIC-fused sarcomas have a shorter overall survival compared to those with BCOR-rearranged sarcomas [1, 3].

Newly diagnosed patients with Ewing sarcomas mostly receive induction chemotherapy containing the combination of anthracyclines, vinca alkaloids, and alkylating agents such as vincristine, ifosfamide, doxorubicin, and etoposide (VIDE). Additionally, other chemotherapeutical drugs are under investigation, such as busulfan and melphalan, and the effect of monoclonal antibody treatment targeting IGFI was also tested [3]. Preclinical experiments suggest that PARP inhibitor treatment can potentiate the effect of chemotherapy or radiotherapy in soft tissue sarcomas, particularly with gene fusions EWSR1::FLI1 and EWSR1::ERG [26]. In Ewing sarcoma cell lines, a direct interaction between the EWSR1::FLI1 fusion protein and PARP1 was described. As the fusion protein also promotes the expression of PARP1, these cells were particularly sensitive to PARP inhibition. It was also demonstrated that the PARPi-mediated cytotoxicity in Ewing sarcoma cells is further enhanced both *in vitro* and *in vivo* upon combination with clinically used topoisomerase inhibitors irinotecan and temozolomide [27]. In another study, they found in murine models that irinotecan potentiated the cytotoxic effect of PARP inhibitors in a much lower dose than temozolomide and had a better tolerability [23].

Putative RNA/DNA helicase SLFN11 is strongly expressed in Ewing sarcomas. SLFN11 expression is positively correlated with sensitivity to DNA-damaging agents including topoisomerases in a variety of cancer types [28]. It was found that EWS-FLI1 enhances SLFN11 expression in ES cells and SLFN11 expression was associated with increased tumor-free survival in ES patients. SLFN11 also sensitized the cells to combined treatment with PARP and topoisomerase inhibitors [29]. Gartrell et al. described that the protein level of SLFN11 in pediatric sarcomas was present in 70% of the cases, although at variable levels. In cell lines, sensitivity to the PARP inhibitor talazoparib and the topoisomerase I inhibitor irinotecan correlated with SLFN11 expression levels. However, in patients, higher expression levels in the tumor showed no correlation with favorable outcome [30].

As single agents, the PARP inhibitor olaparib elicited no significant response in patients with advanced Ewing sarcoma who had progressed after standard chemotherapy [31]. Similarly, irinotecan treatment alone had only a modest response in a phase II study in newly diagnosed ES patients with metastatic disease [32]. However, a combination of talazoparib with irinotecan and temozolomide in a phase I trial showed a higher response rate than chemotherapy alone and the best response showed correlation with SLFN11 expression [33].

A number of Ewing sarcoma cell lines carrying the defining EWSR1::FLI1 fusion have already been established from both tumor and pleural effusion samples [8, 34]. To the best of our knowledge, this is the first sarcoma cell model with EWSR1::POU2AF3 fusion and provides a unique opportunity to test therapeutic approaches preclinically.

## Material and methods

### Cell culture and reagents

The PF1095 line was derived from a malignant pleural effusion sample. First, the effusion sample was centrifuged for 10 min at 1,200 x *g* at room temperature. The cell pellet was resuspended in DMEM containing 10% FBS (fetal bovine serum) and 1% penicillin/streptomycin and the cells were seeded in a culture flask. Experiments were started after eight passages to make sure the tumor cell culture was free from non-malignant cells. The sample was collected in collaboration with the West German Biobank Essen (WBE) and the patient provided their written consent for biobanking. The study was approved by the Ethics Committee of the University Hospital Essen (#18-8208-BO). Multiplex Cell Line Authentication (Multiplexion, Heidelberg, Germany) based on single nucleotide polymorphisms (SNP) was performed to demonstrate PF1095 unique cell line identity. The sarcoma cell line EW-7 was kindly provided by Prof. Dr. Günther Richter (Department of Pediatric Oncology and Hematology, Charité, Berlin, Germany). MHH-ES-1 cell line was received from the German Collection for microorganisms and cell cultures (DSMZ, Braunschweig, GermanyPF588 mesothelioma cells were established in our laboratory [35]. The H526 small-cell lung cancer cell line was obtained from ATCC. All cell lines were cultivated in DMEM supplemented with 10% FBS and 1% penicillin-streptomycin at 37°C and 5% CO_2_ in a humidified atmosphere.

Irinotecan and olaparib were purchased from MedChemExpress (Monmouth Junction, NJ, United States) and were dissolved in DMSO in 10 mM and 50 mM concentrations, respectively. SN-38 was acquired from Selleck Chemicals (Houston, TX, United States) and dissolved in DMSO at a 50 mM concentration. Vincristine and etoposide were purchased from Biomol (Hamburg, Germany) and dissolved in DMSO at 5 mM and 10 mM concentrations, respectively. Ifosfamide and doxorubicin were obtained from Niomech (Bielefeld, Germany) and from Merck (Darmstadt, Germany), respectively. All chemicals were stored at −80°C in aliquots.

### Proliferation assay

For proliferation assays, 20,000 cells per well (PF1095, MHH-ES1, and EW-7) were plated in 12- and 24-well plates and incubated for 48 h, 96 h, and 168 h. Viable cell number was measured after cells were trypsinized. The standard cell number assay using acridine orange and DAPI staining was performed on the NucleoCounter NC-3000TM system (Chemometec, Allerod, Denmark). For each time point, cell measurement was done in triplicate.

### Chemosensitivity assays

To measure the sensitivity of the cell lines to different drug treatments, total protein amount-based Sulforhodamine B assays were performed. Five-thousand tumor cells per well were seeded on a 96-well plate, using the 60 inner wells. After an incubation of 24 h, the cells were either treated with a single drug or with a combination treatment and incubated for another 72 h. Next, the cells were washed once with PBS and then 6% TCA was added to each well. The plate was stained with SRB dye (Sigma-Aldrich, St. Louis, MO, United States) and excess dye was washed away with 1% acetic acid. To measure the total protein amount, 10 mM Tris puffer was used to dissolve the protein-bound dye. Optical density was read by a microplate reader (PR 3100 TSC, Bio-Rad, Hercules, CA) at 570 nm. The CompuSyn software (ComboSyn, Inc., Paramus, NJ) was used to calculate the IC_50_. All experiments were performed at least three times. Interaction between olaparib and irinotecan or doxorubicin was tested with the same method by treating the cells with both drugs in all combinations. CI (combinatory index) values, indicating synergistic, additive, and antagonistic effect if CI < 1, CI ≈ 1, and CI > 1, respectively, were calculated with CompuSyn software (ComboSyn Inc., Paramus, NJ, United States) according to Chou and Talalay [36].

### Cell cycle analysis

NucleoCounter NC-3000TM system (Chemometec, Allerod, Denmark) and its standard cell cycle analysis protocol and solutions were used. First, 200,000 PF1095 tumor cells were seeded in each well of a 6-well plate. After an incubation of 24 h, they were treated with the drugs for 72 h. Cells were trypsinized and incubated for 5 min at 37°C with the lysis buffer supplemented with DAPI stain solution. Afterwards, stabilization puffer was given to each reaction and cellular fluorescence was detected. To distinguish among the different cell cycle phases, the DNA content of the cells was considered.

### Immunoblot

To isolate proteins, tumor cells were seeded on 6-well-plates. 24 h later, the cells were treated for 24 or 72 h. Afterwards, cells were washed twice with PBS and total protein was precipitated with 6% TCA. Following one-hour incubation at 4°C, the protein was harvested and samples were centrifuged for 10 min with 8,000 rpm at 4°C. The pellet was resuspended in electrophoresis sample puffer (62.5 mM Tris–HCl, pH 6.8, 2% SDS, 10% glycerol, 5 mM EDTA, 125 mg/mL urea, 100 mM dithiothreitol). The proteins were loaded on 7.5%, 10%, and 15% acrylamide gels in equal protein amounts (20 or 30 μg). The immunostaining was performed with the following primary antibodies: anti-PD-L1 (Cell Signaling, E1L3N, 1:1000), anti-EWSR1 (Cell Signaling #11910, 1:1000), anti-POU2F3 (Cell Signaling, E5N2D, 1:100), anti-PARP (Cell Signaling #36135, 1:1000), anti-p53 (Cell Signaling, 7F5, 1:1000), anit-SLFN11 (Cell Signaling, D8W1B, 1:1000), anti-pAKT (Cell Signaling, 193H12, 1:1000), anti-AKT (Cell Signaling, 9272, 1:1000), anti-β-Catenin (Santa Cruz, Sc-7199, 1:500), anti-OCA-T1 (Cell Signaling #20217, 1:1000), anti-pERK (Cell Signaling, 4696, 1:1000), anti-β-Tubulin (Abcam, ab20775), and GAPDH (Cell Signaling, 5174, 1:1000).

HRP- conjugated anti-rabbit antibody (Jackson ImmunoResearch, 1:10,000) was used as a secondary antibody, To develop the film, ECL Western blotting Substrate (Thermo Scientific, Waltham, MS, United States) was used and luminography was performed.

### Flow cytometry

After trypsinization, cells were counted and resuspended in PBS. Fifty thousand cells per well were pipetted into a 96-well plate. After centrifugation (1800 U/min) of the plate, the supernatant was removed and the cells were washed with washing buffer (10% FBS, 1% natriumazid in DPBS). FC blocking solution was applied to reduce non-specific antibody binding (Biolegend, San Diego, CA, United States) and cells were incubated with Ganglioside GD2 antibody (mouse monoclonal antibody with Alexa Fluor 633 conjugate, Cell Signaling, 51133, 1:50) and rabbit (DA1E) mAb IgG XP Isotype Control Alexa Fluor 647 conjugate (BioLegend, 357305) for 15 min at RT. After three washes, cells were fixed with 2% paraformaldehyde solution in DPBS. Flow cytometry was performed with CytoFlex LX (Beckman Coulter). Since the viability in these untreated cultures was above 90%, we did not use any specific live cell dyes. The cell debris and duplets were excluded during the forward scatter/side scatter gating. The isotype control staining was used to establish the gating for GD2 positive cells.

### Immunohistochemical analysis

Formalin-fixed paraffin-embedded cell block was prepared from the PF1095 cells following trypsinization and 4% formaldehyde fixation. Sections of 3 µm thickness were cut and immunohistochemistry was performed for CD99 and PD-L1 by using the Ventana BenchMark Ultra automated staining system (Roche Tissue Diagnostics, Grenzach-Vyhlen, Germany). Subsequent color development was done with OptiView staining kit (Roche Tissue Diagnostics) in the automated staining system, followed by hematoxylin counterstaining.

### Fluorescence *in situ* hybridization (FISH)

A 4 µm section was cut from the PF1095 FFPE cell block and the routine FISH procedure was performed using the ZytoLight^®^ SPEC EWSR1/FLI1 TriCheck™ Probe (ZytoVision, Bremerhaven, Germany).

### Molecular analysis

DNA and RNA was isolated from cultured cells by using DNeasy Blood & Tissue Kit, RNA QIAamp DNA FFPE Tissue Kit, and RNeasy Mini Kit (Qiagen, Hilden, Germany) following the instructions of the manual. We measured DNA and RNA concentrations by using the Qubit^®^ 2.0 Fluorometer assay kits (LifeTechnologies, CA, United States).

For RNA sequencing, cDNA, multiplex amplicons, and a library were prepared with the Archer FusionPlex Sarcoma Panel and MBC Adapter (IDT, Leuven, Belgium). Sequencing was performed at the MiSeq platform (Illumina, San Diego, CA, United States). Up to 24 samples were sequenced within one run (2 × 150 cycles) with a total output of more than 4,5 Gigabases/run. Samples showed a minimum of 50.000 unique fragments.

For DNA sequencing, NGS libraries were created using customized QIAseq-targeted DNA panels (Qiagen), according to the manufacturer’s recommendations. The pooled library was sequenced on MiSeq (Illumina) and analyzed by CLC Genomics Workbench (CLC Bio, Qiagen). Sequencing output was equal to RNA sequencing. The minimum unique molecular identifier (UMI) coverage within a sample was 100x. Mean coverage was higher than 500x. Variants were called with a minimum of 3% variant allele frequency (VAF).

## Results

### Clinical history

**FIGURE 1:**
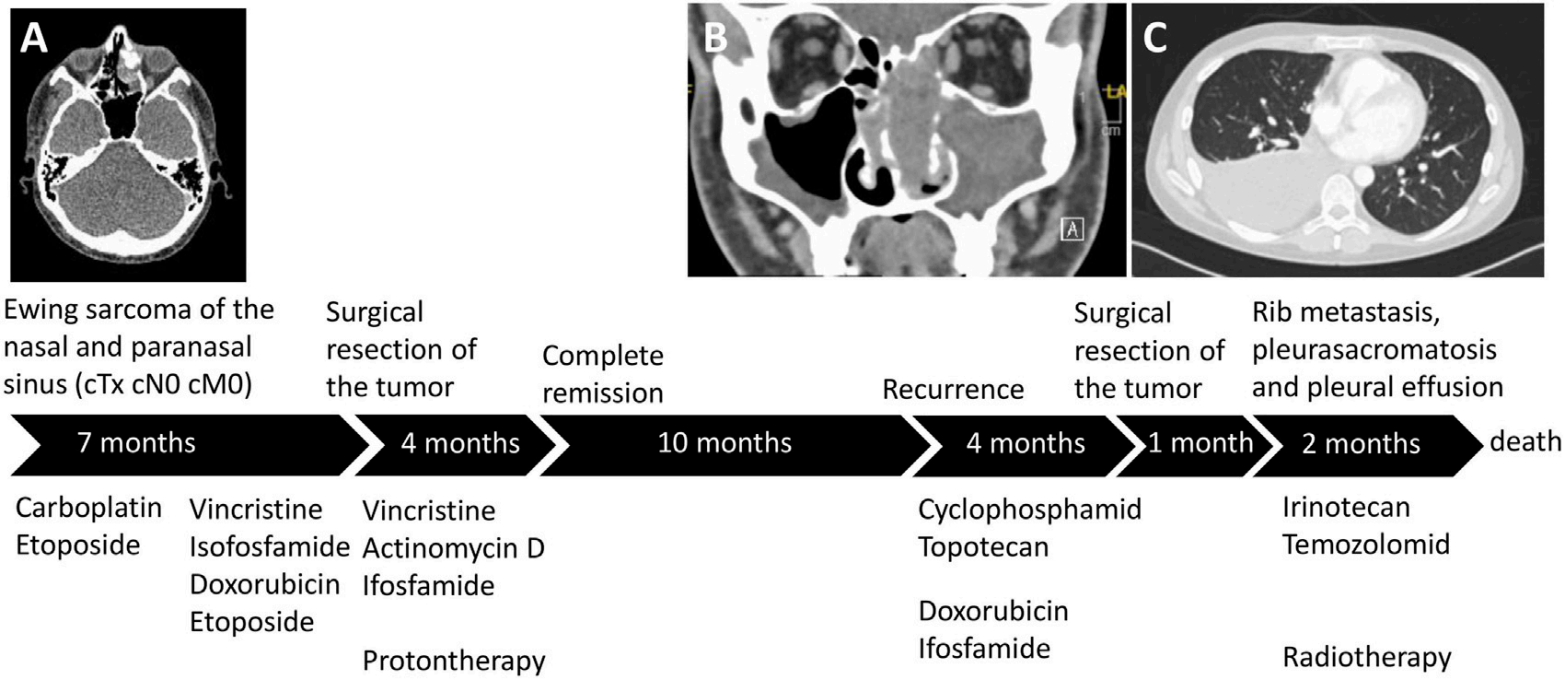
The patient´s treatment course. **(A)** The localization of the primary tumor in the left ethmoidal sinus. After the final diagnosis of Ewing sarcoma, the treatment followed the Ewing 2008 protocol and proton therapy was also performed. **(B)** Following complete remission, the recurrent tumor emerged in the nasal cavity and left maxillary sinus. **(C)** Shortly after the resection of the recurrent tumor, pleurasarcomatosis, rib metastasis, and malignant pleural effusion was detected.

A 23-year-old male patient was initially diagnosed with a poorly differentiated neuroendocrine carcinoma in the left nasal cavity and therefore a carboplatin and etoposide combination therapy was applied in another medical center (Figure 1). After reviewing the tumor´s histology, the diagnosis was altered to Ewing Sarcoma and the patient received treatment following the Ewing 2008 protocol including an induction therapy with vincristine, ifosfamide, doxorubicin, and etoposide. Following the surgical R0 resection of the tumor adjuvant, vincristine, actinomycin D, and ifosfamide therapy was administered. In addition to chemotherapy, the patient received local radiotherapy with protons. As a result of this treatment strategy, the patient had a complete remission. Twenty-one months after the initial diagnosis, local recurrence was observed. Cyclophosphamide and topotecan treatment elicited a partial remission but, due to progression, the treatment regimen of doxorubicin and ifosfamide was introduced and the recurrent tumor was resected. One month later, a metastasis in the right ninth rib and pleural effusion was discovered. The patient received irinotecan and temozolomid treatment and palliative radiation. However, pleurasarcomatosis was diagnosed and the cell line was established from the malignant pleural effusion. The patient succumbed to the disease 28 months after initial diagnosis.

The primary tumor was weakly positive to EMA and synaptophysin staining but it showed high expression of CD56. The tumor cells were negative to CK7 chromogranin, CD3, CD20, and LCA, but they had a high proliferation index. Based on the morphological characteristics and the staining results, the tumor was classified as low-differentiated neuroendocrine carcinoma. Reference pathological analysis altered the diagnosis to Ewing sarcoma based on FISH analysis that revealed an EWSR1 translocation with an unknown partner. All the tumor cells showed membrane positivity to CD99. Additionally, 60% of the cells in the tumor expressed disialoganglioside GD2 at moderate level and 10% at high level.

### Characterization of the PF1095 cell line

**FIGURE 2:**
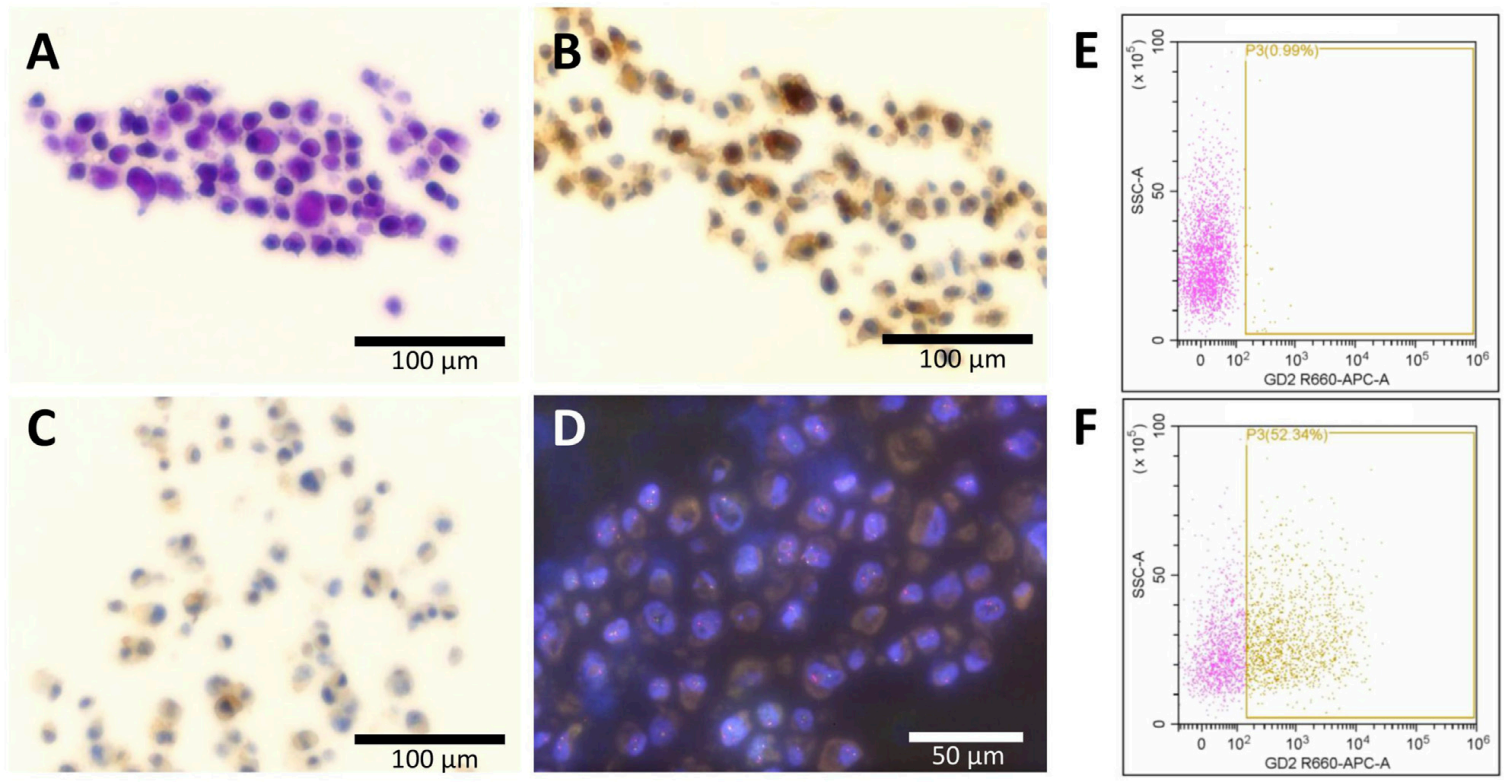
Expression of histopathological markers in the PF1095 cells. Cell blocks were analyzed by **(A)** hematoxylin eosin, **(B)** CD99, and **(C)** PD-L1 staining as well as by break-apart FISH analysis of the EWSR1 gene **(D)**. Cell surface expression of disialoganglioside GD2 was analyzed by flow cytometry, with isotype control **(E)** or with anti-GD2 primary antibody **(F)**, and 50% of the cells showed positive staining.

First, we analyzed the expression of the pathological markers described in the tumor tissue in the PF1095 cells (Figure 2). We found that the cells were strongly positive to CD99 and showed sporadic positivity to CD56 while p53 staining was negative. We further investigated the cell surface expression of disialoganglioside GD2 by flow cytometry and found that around 50% of the tumor cells were positive, similarly to the tumor tissue. Interestingly, we found PD-L1 expression in PF1095 cells. FISH analysis proved the presence of a break in the EWSR1 gene (Figure 2D). RNA sequencing identified POU2AF3 as the EWSR1 fusion partner (EWSR1 Exon 15 - POU2AF3 Exon 2). DNA sequencing revealed a frameshift deletion in the TP53 gene (c.277dupC). This specific TP53 mutation was described as a germline alteration in a Chinese cohort of hereditary breast cancer patients [37], however, we could not detect the mutation in tumor-free tissue.

**FIGURE 3:**
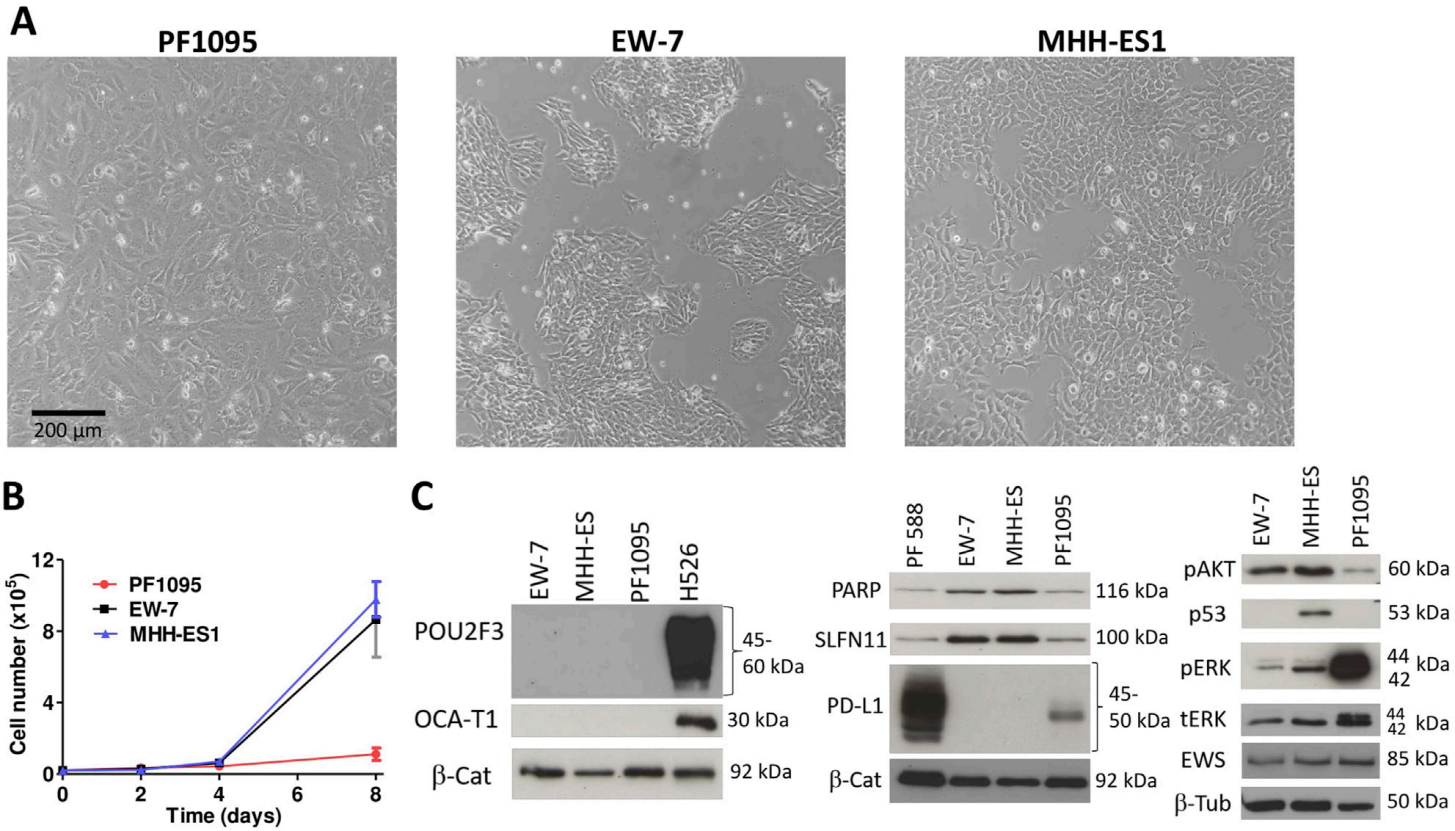
Characterization of PF1095 cells in comparison with ES cell models. **(A)** Cell morphology, phase contrast images (×20 objective). **(B)** Proliferation rate. Cell number was determined after 2, 4, and 8 days. Bars represent means ± SEM from three independent experiments. **(C)** Baseline protein expression was analyzed by Western blot. Pictures show one representative experiment of two or three independent measurements. β-Tubulin and β-Catenin proteins were used as loading controls.

Next, we characterized the morphology, the proliferation rate, and the baseline expression of a panel of proteins of the PF1095 cells in comparison with two Ewing sarcoma cell models. PF1095 cells did not show the small, rounded cell morphology of Ewing sarcoma cells (Figure 3A) and their proliferation rate was also considerably slower (Figure 3B). Immunoblot analysis recapitulated the result of the cell block immunohistochemistry that p53 was not expressed in PF1095 cells but PD-L1 protein was present (Figure 3C). In comparison with the Ewing sarcoma cell lines, EWS protein was strongly expressed in all cell lines, while pAKT, PARP, and SLFN11 levels were lower in the PF1095 cells than in the two Ewing sarcoma S cell lines. Interestingly, ERK activation was extremely high in PF1095. POU2AF3 (COLCA2) was described as a coactivator of POU2F3 in the cells and we used a small-cell lung cancer cell line (H526) as a control. However, we could not detect POU2F3 protein in the cells. POU2AF2 (OCA-T1) is also a coactivator of POU2F3 but we could not detect OCA-T1 in the cells. Unfortunately, there is no commercially available antibody against POU2AF3 (COLCA-2).

### Drug sensitivity of the PF1095 cell line

**FIGURE 4:**
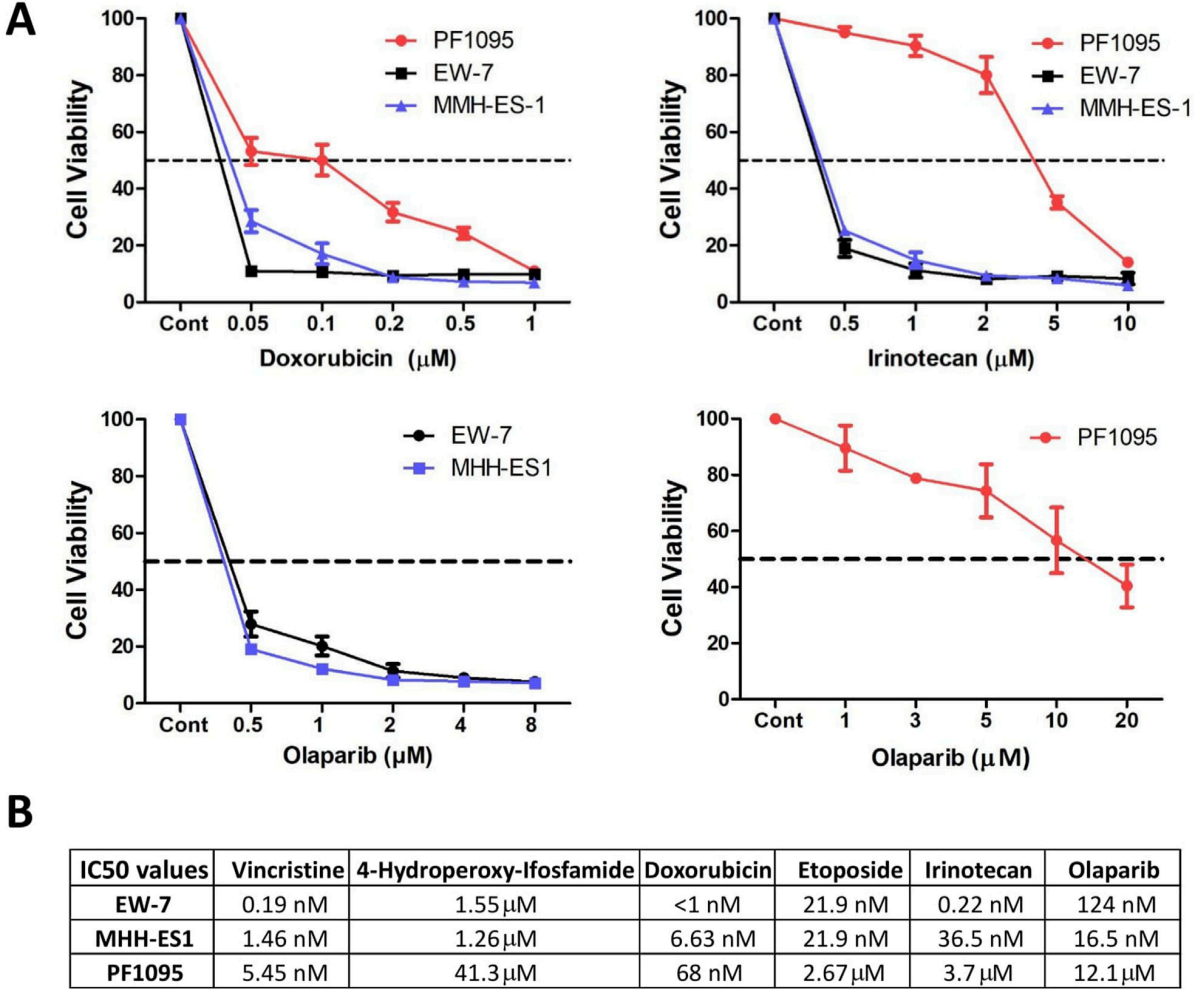
Drug sensitivity of the PF1095 cell line in comparison with the Ewing sarcoma cell models. **(A)** Cells were treated with increasing amount of doxorubicin, irinotecan, and olaparib for 72 h. Cell viability was determined by Sulforhodamine B assay. Bars represent means ± SEM from three independent experiments. **(B)** Table with the IC50 values for vincristine, ifosfamide, doxorubicin, etoposide, irinotecan, and olaparib determined by cell viability assays.

As VIDE protocol is the standard of care for Ewing sarcomas, we analyzed the sensitivity of PF1095 cells to the drugs included in this combination (vincristine, ifosfamide, doxorubicin, and etoposide) and to irinotecan and the PARP inhibitor olaparib (Figure 4A; Supplementary Figure S2). We found that the PF1095 cell line was much less sensitive to ifosfamide, etoposide, irinotecan, and olaparib than the two Ewing sarcoma cell lines, EW-7 and MHH-ES-1 (Figure 4B). In case of vincristine and doxorubicin, the PF1095 cells were sensitive to both treatments with an IC50 value of 5.45 nM and 68 nM, respectively, but still less sensitive than the two Ewing sarcoma cell lines.

### Combination treatments with PARP inhibitor olaparib

**FIGURE 5:**
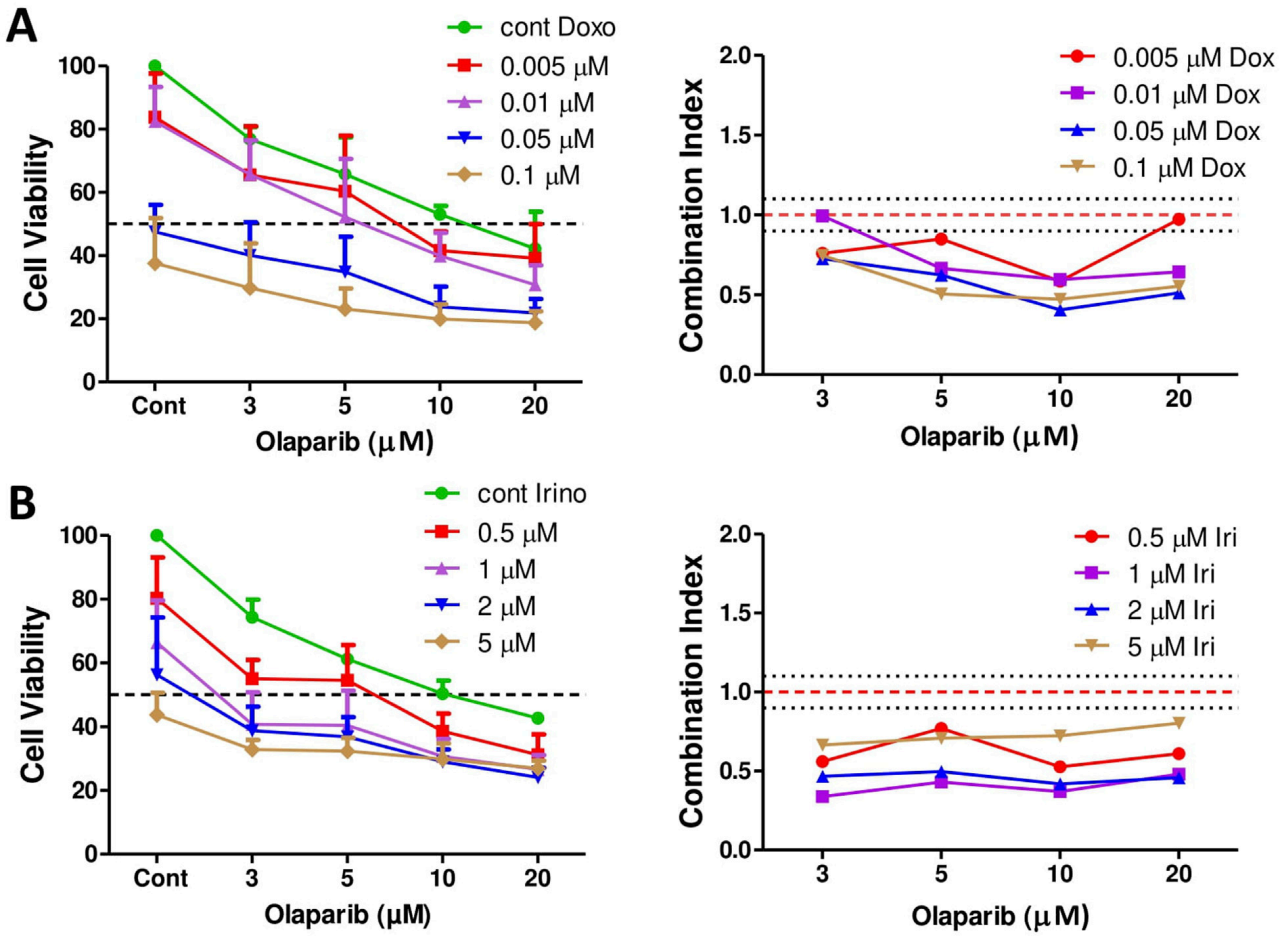
The effect of combination treatments on cell viability. Combination of doxorubicin and olaparib **(A)** and irinotecan and olaparib **(B)** were analyzed by Sulforhodamine B assay. Cells were treated with increasing amount of doxorubicin or irinotecan and olaparib for 72 h. Combination index (CI) was calculated for each combination and indicates synergism (CI < 0.9), additive effect (CI is between 0.9 and 1.1), or antagonism (CI > 1.1). CI was calculated with the CompuSyn software. Dotted lines show these cut-offs.

It was demonstrated previously that Ewing sarcoma tumor cells are particularly sensitive to PARP inhibition *in vitro* [27]. Accordingly, we investigated if a combination treatment with olaparib potentiates the effect of doxorubicin or irinotecan treatment in PF1095 cells. We found that in case of both combinations there was a strong synergistic effect and combination treatments further reduced cell viability (Figure 5).

**FIGURE 6:**
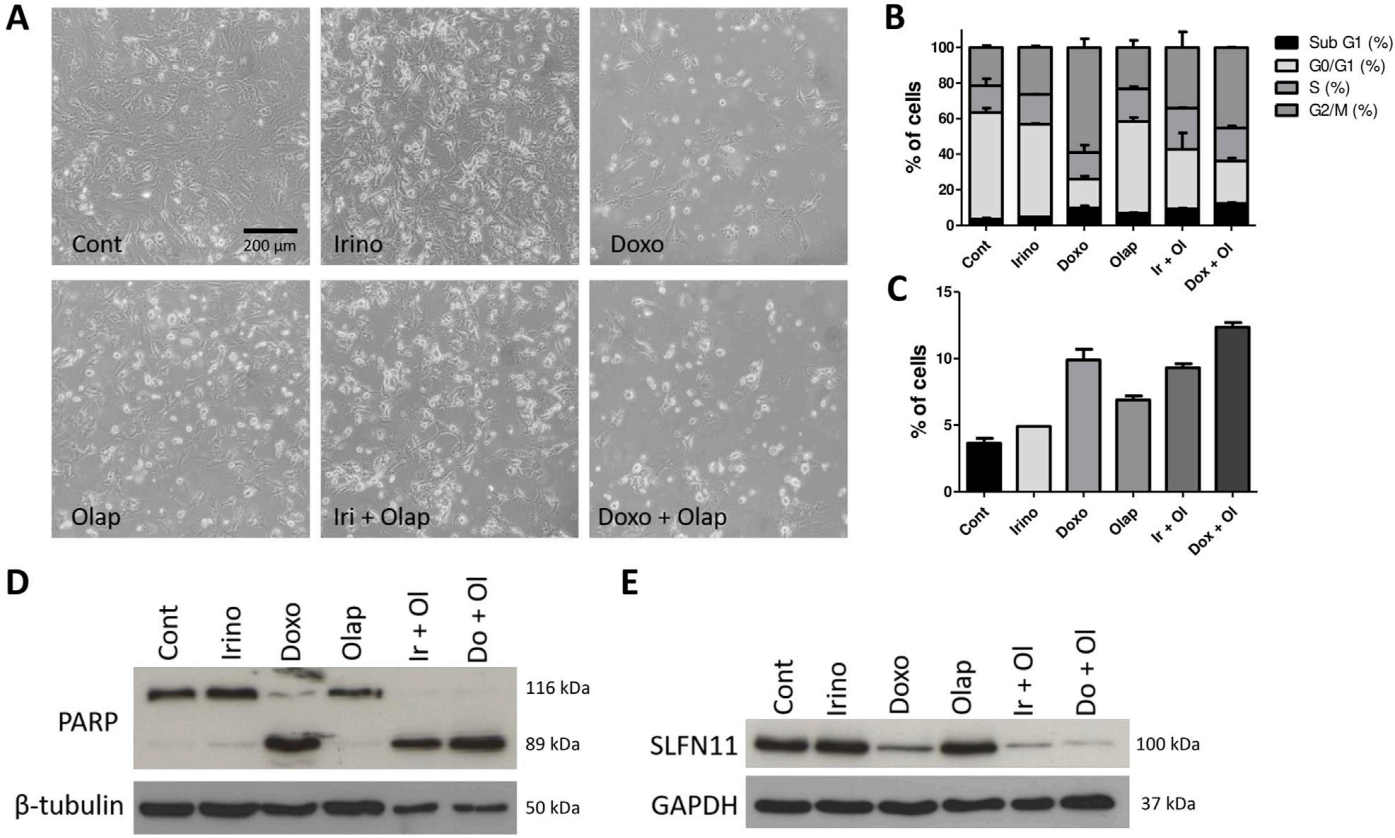
Combination treatments induce cell death and decreased SLFN11 expression. **(A)** Representative phase contrast images of PF1095 cells after 72 h’ treatment with irinotecan (1 μM), doxorubicin (0.05 μM), olaparib (5 μM) alone and in combinations (×20 objective). **(B)** Cell cycle analysis with all cell cycle phases and **(C)** the ratio of the cells in the subG1 phase. Bars represent means ± SEM from three independent experiments. **(D, E)** PARP and SLFN11 proteins were analyzed by Western blot. Pictures show one representative experiment of two or three independent measurements. β-Tubulin and GAPDH proteins were used as loading controls.

We further investigated the effects of these combination treatments in PF1095 cells and performed cell cycle assays and protein analysis (Figure 6). Cell cycle analysis showed that all treatments increased the ratio of cells in the subG1 phase and both the irinotecan-olaparib and the doxorubicin-olaparib combinations increased cell death significantly compared to the single treatments. Both irinotecan and doxorubicin treatments initiated G2M cell cycle arrest in the cells (Figures 6A–C). In good accordance with these results, protein analysis showed that the apoptotic fragment of the PARP protein was generated in the cells after treatment with both combinations. As a single treatment, neither irinotecan nor olaparib initiated PARP cleavage substantially, however, in the combination only the cleaved form of the PARP protein was detected. Doxorubicin as a single treatment already initiated the formation of the apoptotic fragment (Figure 6D). Previous studies showed that the expression level of SLFN11 protein positively correlates with sensitivity to DNA damaging agents like topoisomerase inhibitors, alkylating agents, or DNA synthesis inhibitors [28] and with sensitivity to PARP inhibitors as well [29]. We showed above that SLFN11 is expressed in PF1095 cells, although in lesser amounts than in the Ewing sarcoma cell lines. We found that treatment with doxorubicin and, particularly, both combination treatments reduced SLFN11 expression in PF1095 cells (Figure 6E).

## Discussion

We established a novel sarcoma cell line, PF1095, with a EWSR1::POU2AF3 fusion. Very recently, sarcomas with this specific gene alteration were proposed to be a novel entity as they have unique clinical characteristics [16]. They often originate from the head and neck region, especially from the sinonasal track, are prone to early metastasis, and are coupled with particularly bad prognoses. In good accordance with these findings, the primary tumor in the patient localized in his left nasal cavity, showed positivity for both neuroendocrine (synaptophysin, CD56) and sarcoma markers (CD99), and had a high proliferation index. Later, an EWSR1 translocation was detected with an unknown partner and we identified POU2AF3 as a fusion partner in the PF1095 cells. In this cell line, a new fusion between EWSR1 exon 15 and POU2AF3 exon 2 was identified. Previously, fusions of EWSR1::POU2AF3 were reported twice with an exon 9–exon 2 breakpoint and once with an exon 10–exon 2 breakpoint. As an additional mutation, a frameshift deletion in the TP53 gene was found in PF1095 cells. In the previously described EWSR1::POU2AF3 fusion sarcomas, RAD51, NOTCH1, and CDKN2A loss was found. The disease course was similar to some of the earlier cases, as the patient in our study first responded well to the Ewing protocol treatment in combination with surgery, however, after recurrence, distant metastasis developed despite further treatment and the patient succumbed to the disease 28 months after initial diagnosis.

Importantly, both in the tumor tissue and in the cell line, around 50% of the cells expressed cell surface disialoganglioside GD2. This carbohydrate-containing sphingolipid is highly expressed in several cancer types such as neuroblastomas, melanomas, and, in certain cases, in Ewing sarcomas and small-cell lung cancers. Since its expression in normal tissues is limited, it became a target of cancer therapy; anti-GD2 monoclonal antibodies, disialoganglioside GD2 vaccines, antibody–drug conjugates, and several other new approaches are under development. The anti-GD2 monoclonal antibody, dinutuximab, has been approved for high-risk neuroblastoma in pediatric patients [38, 39].

We also found that PF1095 cells express PD-L1, a target of immune checkpoint inhibitor therapies. Ewing sarcomas are usually immunologically cold due to low tumor burden and an immune suppressive tumor microenvironment; in addition, PD-1 or PD-L1 expression is only present in 20% of patients. Accordingly, in Ewing sarcomas and osteosarcomas, immune checkpoint therapies showed low efficacy clinically [40]. However, in a rare soft-tissue sarcoma, namely in alveolar soft part sarcoma, PD-L1 expression rate is higher, and anti–programmed death ligand 1 (PD-L1) agent atezolizumab elicited sustained response in one-third of the patients in a phase-2 study [41]. Based on our observations, the analysis of GD2 and PD-L1 expression might be worthwhile in POU2AF3 fusion sarcomas and should be tested in a clinical setting in this otherwise difficult-to-treat malignancy.

The distinct small and round cell morphology of Ewing sarcoma cells is not present in the PF1095 cells, which are larger and have a more elongated cell shape. Using immunoblot analysis, we could not detect the transcription factor POU2F3 protein in the cells, even though POU2AF3 was determined as a coactivator of this protein in tuft cells. The other coactivator of POU2F3, OCA-T1, which was described to be expressed in a mutually exclusive manner with POU2AF3, was also not present in the cells. These results suggest that the tumor most likely does not originate from tuft cells and the EWSR1::POU2AF3 fusion protein drives the tumor growth through other pathways.

We compared the proliferation rate and the activation of some major signaling pathways between the PF1095 cell line and two Ewing sarcoma cell lines. We found that both the proliferation rate and Akt activation was lower in the PF1095 cells, however, surprisingly, ERK activation was extremely high in comparison with the Ewing sarcoma cells. Total ERK expression was also higher in PF1095 cells. It has been suggested that, in bone sarcomas, MAPK inhibition has some clinical benefit as it can reduce proliferation and invasiveness of tumor cells [42]. A recent study showed that MAPK signaling and the expression of anti-apoptotic protein MCL1 is elevated in osteosarcoma lung metastases [43]. Further analysis of the MAPK signaling in POU2AF3 fusion sarcomas might also be interesting.

SLFN11 expression is a known predictor of sensitivity to DNA-damaging agents and it is usually abundantly present in Ewing sarcoma cells [29]. We found that SLFN11 is expressed in the PF1095 cells but at a much lower level than in the two Ewing sarcoma cell lines. We also analyzed the relative sensitivity of the three cell lines to the agents used in the VIDE protocol and found that the PF1095 cells were less sensitive to all four drugs (vincristine, ifosfamide, doxorubicin, and etoposide) and also to irinotecan. All these drugs were used during the treatment of the patient and prior to the establishment of the cell line. It is important to note that the cell line was established at a very late time point in the disease course, after the formation of the pleural metastasis.

It was previously described that the chimeric transcription factor EWSR1::FLI1 is a positive regulator of SLFN11 expression and SLFN11 is necessary for a response to the combination treatments of the alkylating agent temozolomide with topoisomerase I or PARP inhibitors [29]. It was also suggested that EWSR1::FLI1 promotes the expression of PARP1 and makes cells harboring this mutation particularly sensitive to PARP inhibitors [27]. We compared the expression level of PARP and the sensitivity of the cells to the PARP inhibitor olaparib and found that baseline PARP level was lower in PF1095 cells than in the two Ewing sarcoma cell lines. Accordingly, the sensitivity of the PF1095 cells to olaparib was almost one magnitude lower than the Ewing sarcoma cells, which had a very high sensitivity. Still, when we applied olaparib in combination with either doxorubicin or irinotecan there was a strong synergistic effect. Both combinations induced cell death and G2M cell cycle arrest. Doxorubicin as a single treatment already had these effects, but they were further increased by the combination. However, as a single agent, neither irinotecan nor olaparib induced cell death or major cell cycle alteration. Importantly, both combination treatments and doxorubicin treatment alone reduced SLFN11 expression, which was described as a factor in acquired chemoresistance in small-cell lung cancer patients [44].

In summary, our results show that the PF1095 cell line is an invaluable tool to better understand EWSR1::POU2AF3 sarcomas and to test potential new combination therapies that might increase therapeutic efficacy.

## Data Availability

The raw data supporting the conclusions of this article will be made available by the authors, without undue reservation.
