# Supplementary material for: Characterization of a novel sarcoma cell line with an EWSR1::POU2AF3 fusion
Source: Pathol Oncol Res. 2025 Mar 11;31:1611986. doi: 10.3389/pore.2025.1611986 (PMC11932835; doi:10.3389/pore.2025.1611986)
Supplement: Supplementary file 1 [file DataSheet1.PDF]

**Supplementary Data**

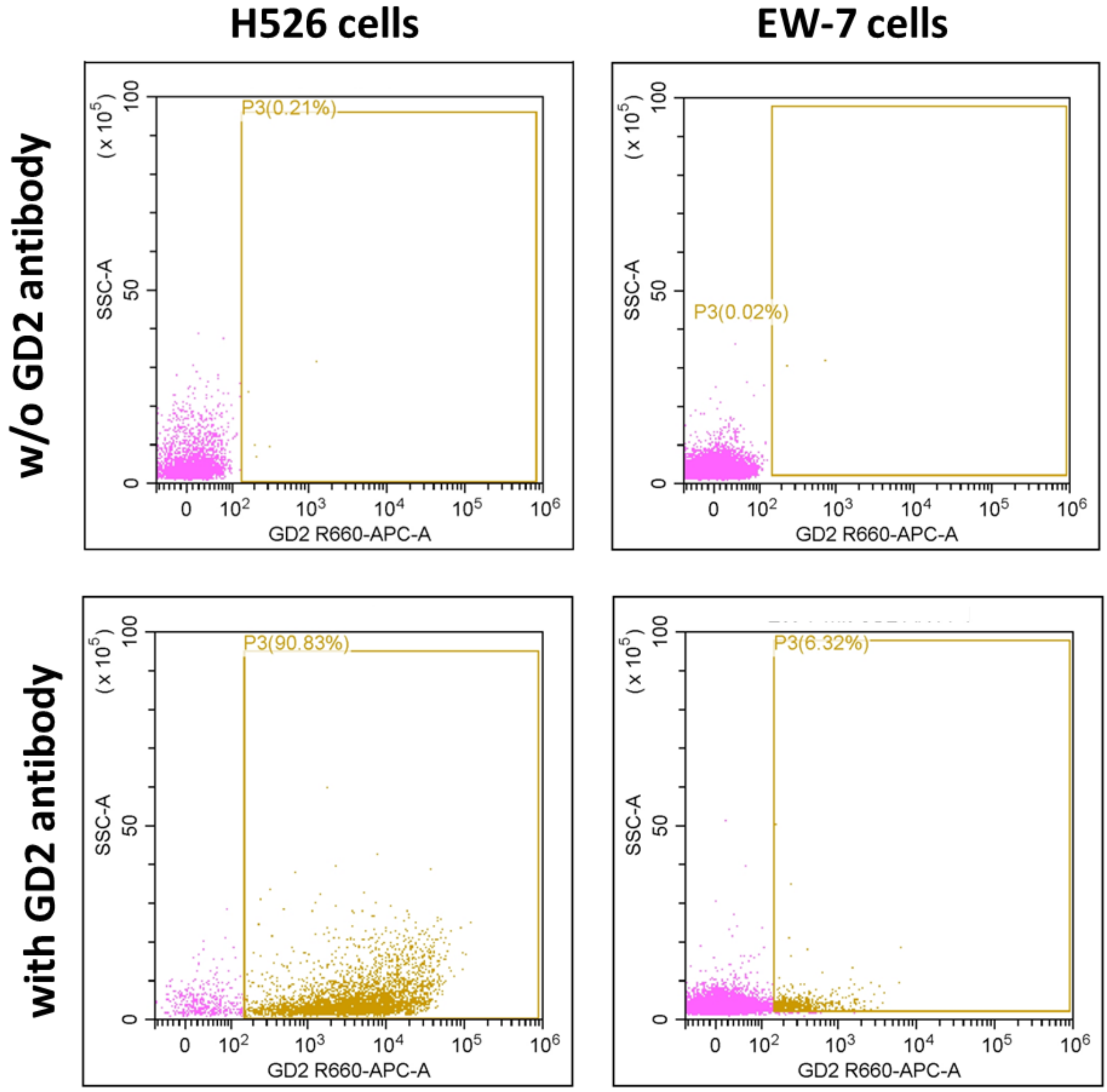


**Supplementary figure 1.** Flow cytometry analysis of GD2 expression. H526 cells served as positive controls. EW-7 cells showed only very limited expression of GD2 on the cell surface.

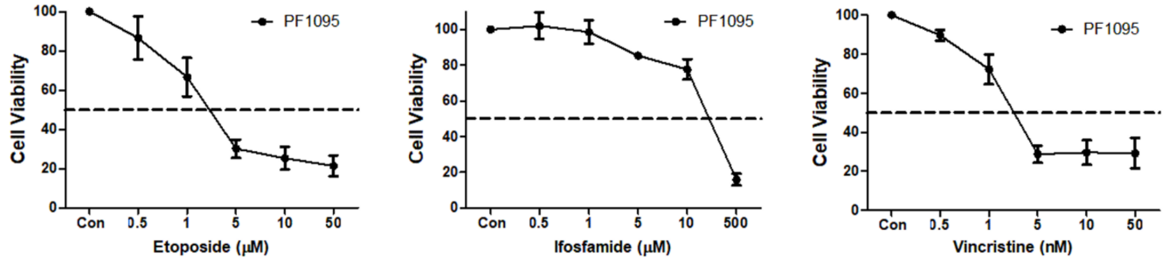


**Supplementary figure 2.** Drug sensitivity of the PF1095 cell line. (A) Cells were treated with increasing amount of etoposide, vincristine and ifosfamide for 72 hours. Cell viability was determined by Sulforhodamine B assay. Bars represent means ± SEM from three independent experiments.

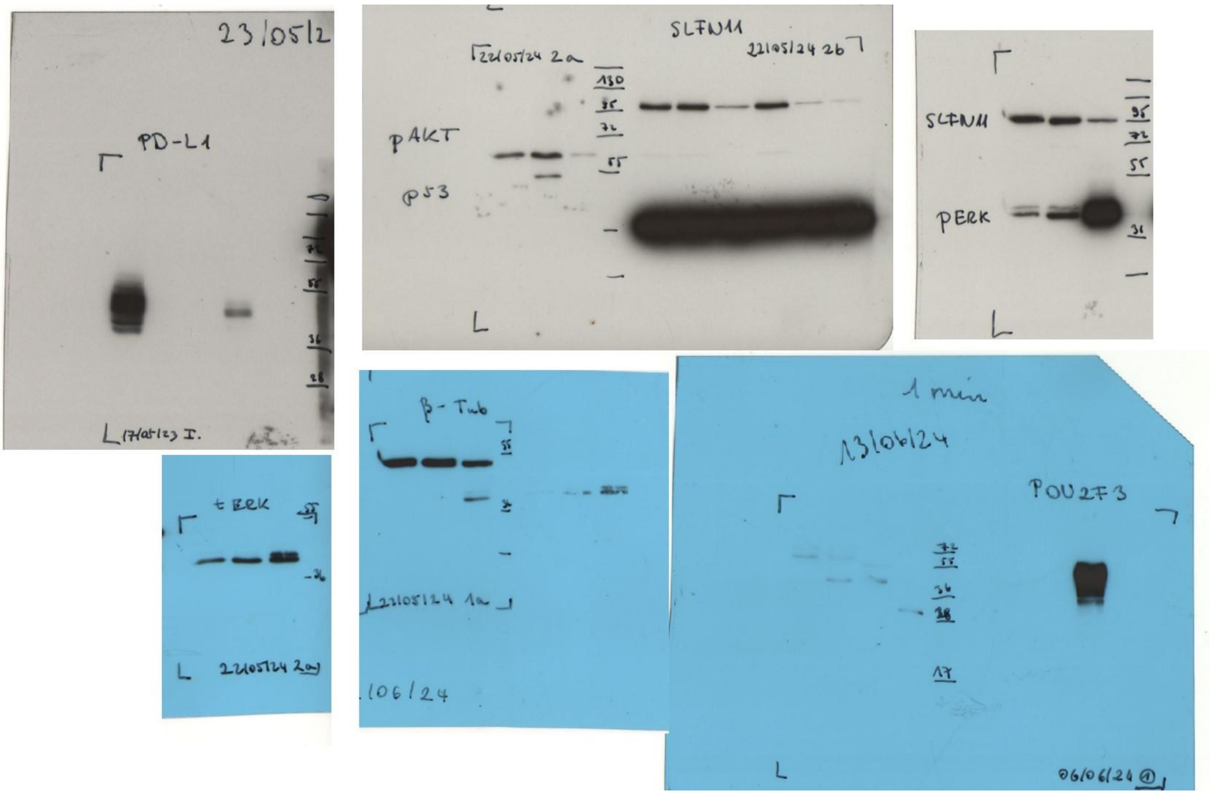


**Supplementary figure 3.** Original western blots from which the corresponding bands were cropped in figure 3. The bands of the molecular weight marker were marked on the X-ray films to verify band size.

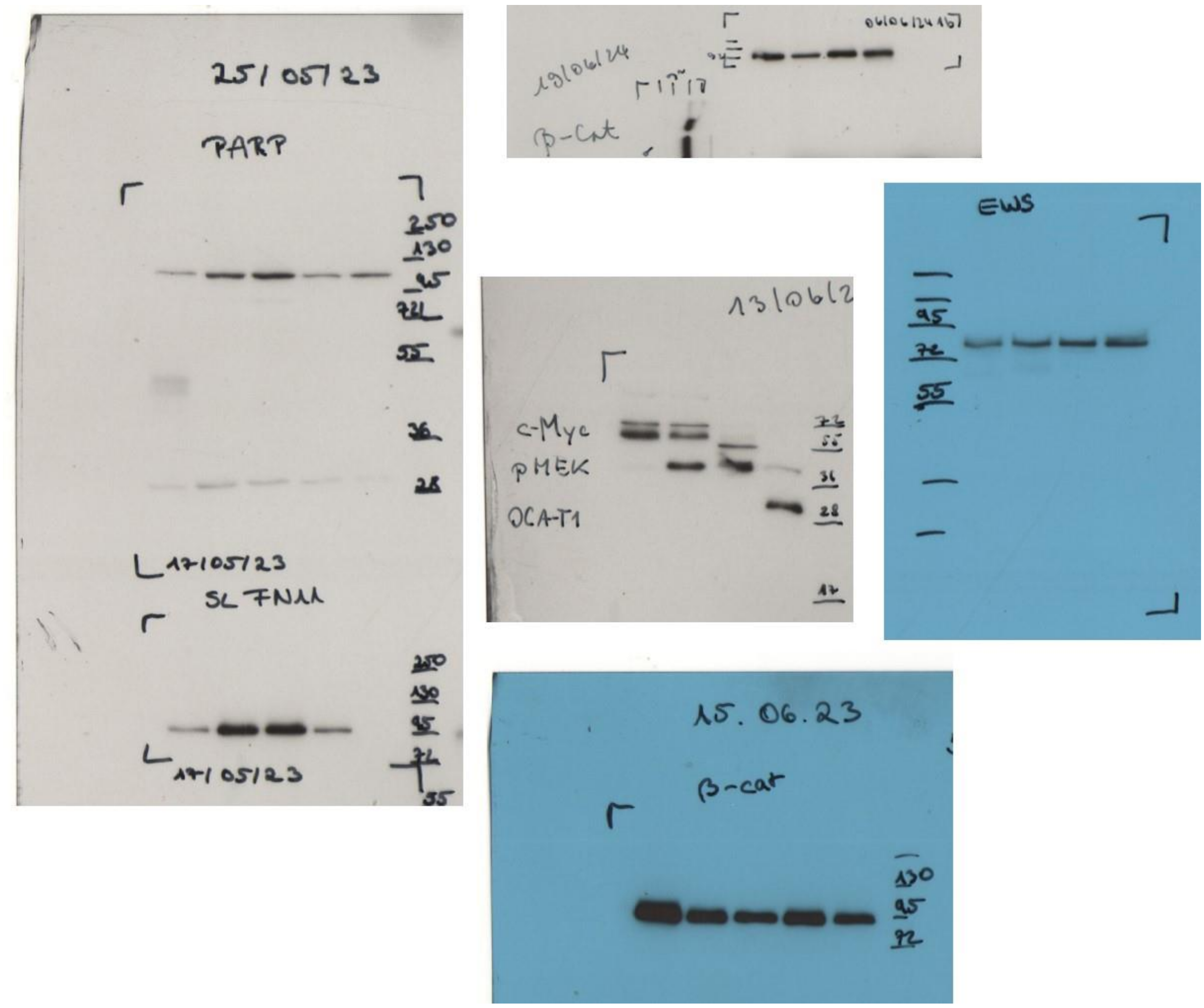


**Supplementary figure 4.** Original western blots from which the corresponding bands were cropped in figure 3. The bands of the molecular weight marker were marked on the X-ray films to verify band size.

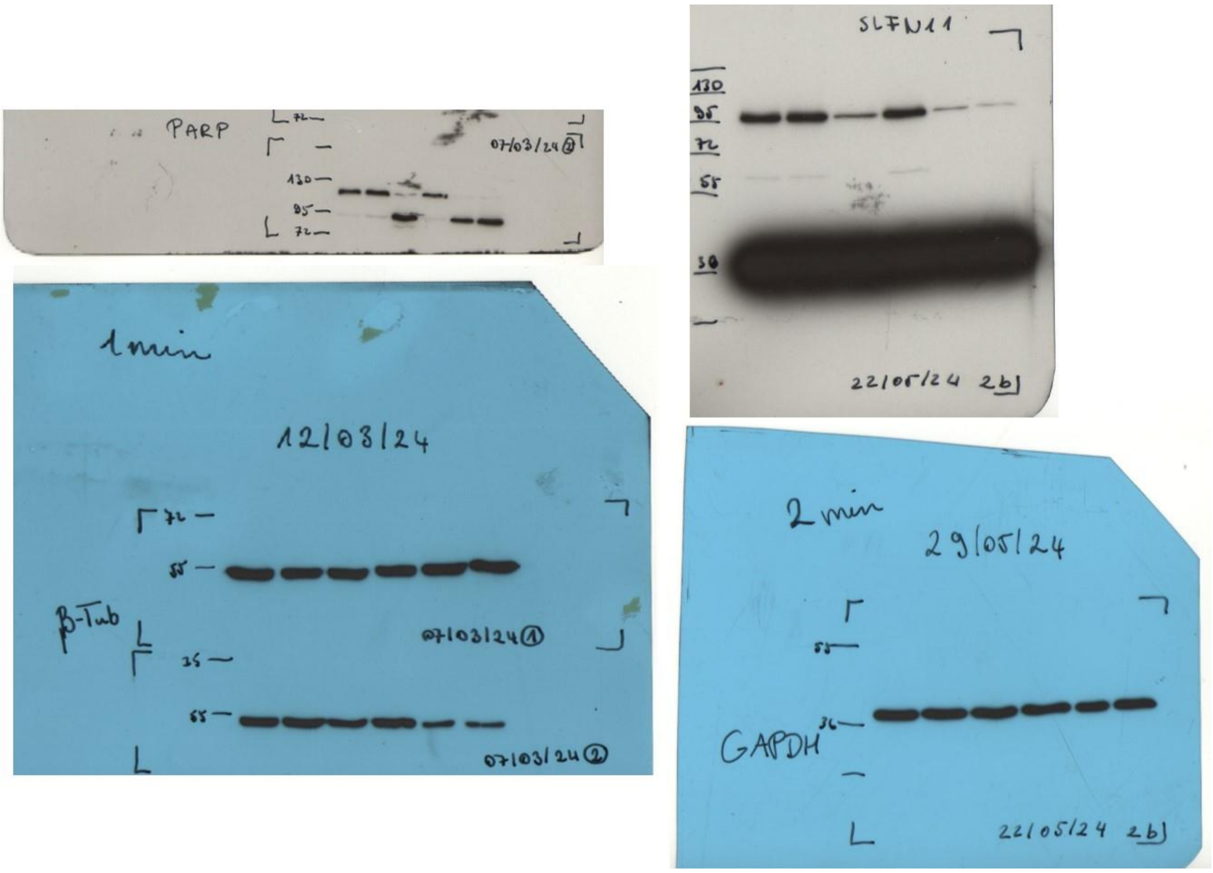


**Supplementary figure 5.** Original western blots from which the corresponding bands were cropped in figure 6. The bands of the molecular weight marker were marked on the X-ray films to verify band size.
